# Serum Total Bilirubin and Progression of Chronic Kidney Disease and Mortality: A Systematic Review and Meta-Analysis

**DOI:** 10.3389/fmed.2020.00549

**Published:** 2021-01-25

**Authors:** Jia Li, Dongwei Liu, Zhangsuo Liu

**Affiliations:** ^1^Department of Nephrology, The First Affiliated Hospital of Zhengzhou University, Zhengzhou, China; ^2^Research Institute of Nephrology, Zhengzhou University, Zhengzhou, China; ^3^Key Laboratory of Precision Diagnosis and Treatment for Chronic Kidney Disease in Henan Province, Zhengzhou, China; ^4^Core Unit of National Clinical Medical Research Center of Kidney Disease, Zhengzhou, China

**Keywords:** serum total bilirubin, chronic kidney disease, disease progression, mortality, meta-analysis

## Abstract

**Background:** Previous studies have suggested that serum total bilirubin (STB) levels are associated with heightened chronic kidney disease (CKD) and mortality in both the general population and nephropathy patients. However, these results remain inconsistent. The aim of our study was to investigate whether STB was a predictor for progression of CKD and mortality by meta-analysis.

**Methods:** We performed a systematic literature search in PubMed, Web of Science, MEDLINE, EMBASE, Google Scholar, and Cochrane Library's database up to June 30, 2019. Pooled risk ratios (RR) and corresponding 95% confidence intervals (CI) were extracted for the highest vs. lowest category STB levels within the physiological range, and a random-effects model was applied to calculate the dose–response relationships. A pooled hazard ratio (HR) was used to investigate the association between STB levels and mortality in dialysis patients.

**Results:** A total of 16 studies, wherein participants were followed from 21 months to 7 years, were eligible for inclusion in the study. For the categorized STB, 11 studies with 41,188 participants were identified and analyzed. Patients with the highest STB levels were associated with a lower risk of CKD (RR = 0.64; 95% CI 0.55–0.73) compared to those with the lowest STB levels. Furthermore, based on seven studies, a pooled RR of 0.89, 95% CI (0.80–0.99) was observed for the continuous STB levels (per 0.2 mg/dL increase). Four studies that included 51,764 participants illustrated that there was no association between STB levels and all-cause mortality (HR = 0.77; 95% CI 0.42–1.41). A prominent negative linear relationship (X^2^ = 14.70; *P* = 0.0001) was found between STB levels and risk of CKD. Subgroup analyses showed that there were no significant differences in the subgroup adjustment factor except for sample size.

**Conclusions:** Elevated STB levels within a physiological range are associated with lower risk of CKD regardless of the study characteristics and coincide with a liner dose–response relationship. However, whether high STB levels are a protective factor against mortality remains inconclusive. Large-scale randomized controlled trails are needed to target STB levels for predicting renal outcomes.

## Introduction

Chronic kidney disease (CKD), also called chronic kidney failure, is on the rise, and it has become one of the most common complications worldwide. It not only increases the risk of cardiovascular disease and can often progress to end-stage renal disease (ESRD), but it is also related to premature mortality. According to the 2013 Global Burden of Disease Study, CKD ranked 36th in the causes of total number of global deaths in 1990, but rose to 19th in 2013 (1). Much of the general population is admitted to hospital with chronic kidney disease, so early detection, diagnosis, and intervention of adverse factors that may lead to kidney disease are essential for improving the prognosis of patients with CKD. It should also be noted that a rising prevalence of comorbidities and risk factors, such as hypertension, diabetes mellitus glomerulonephritis, and infectious diseases, are also contributing to the high burden of CKD (2). However, these factors do not fully explain the variation and heterogeneity in the prevalence of CKD.

Recently, several studies have demonstrated that oxidative stress can play a crucial role in the pathogenesis of CKD (3–5). Oxidative stress imbalance is generally caused by an overproduction of reactive oxygen species (ROS) or a deficiency of the antioxidant reagent. Bilirubin is a heterogeneous group of antioxidants which derives from the heme catabolism through a complex sequence of reactions. It exists in two forms of serum, direct bilirubin and indirect bilirubin, and both are newly recognized as antioxidant, anti-inflammatory molecules under physiological conditions (6). However, the role that serum total bilirubin (STB) plays within the physiological range in the development and progression of kidney disease remains controversial. Several studies have suggested that an inverse association between STB and the progression of ESRD (7–10) plays a potential protective role in renal outcomes. A large study of the Korean population that was recently published demonstrates that individuals with higher bilirubin levels have a reduced prevalence of CKD originating from diabetes in women (11). Two studies revealed that elevated bilirubin levels have a reduced risk of progressing from urinary microalbuminuria to macroalbuminuria, as well as improved eGFR in diabetic patients (12, 13). Furthermore, Fukui et al. reported that higher circulating serum bilirubin levels were associated with reduced risk of cardiovascular disease and mortality in dialysis patients (14). Although most investigations indicate a potential beneficial effect of bilirubin on renal prognosis, there is some evidence that suggests an inconclusive relationship between bilirubin and clinical endpoints. Wang et al. noted that lower STB was not an independent protective factor in kidney disease progression among hypertensive patients who never smoke (15). Targher et al. demonstrated that higher STB levels were significantly associated with lower eGFR in both non-diabetic and diabetic individuals in unselected outpatients (16). Ryu et al. proved that neither STB nor indirect bilirubin levels were associated with the incidence of CKD (17). Additionally, it was noted that in patients with ESRD who were undergoing hemodialysis, high concentrations of bilirubin were correlated with a higher mortality rate (18). These results indicate the need for evaluating the role of STB levels on the progression of CKD and mortality.

Keeping in mind the unclear interactions between STB levels and the impact of renal outcomes, we conducted this systematic review and meta-analysis to determine whether STB independently contributes to the progression of CKD in both the general population and nephropathy patients. We also evaluate the association between STB and mortality in those who were undergoing regular dialysis and investigated the possibility of it acting as a novel biological factor to predict kidney disease progression.

## Materials and Methods

### Search Strategy

The meta-analysis was conducted according to the checklist of Preferred Reporting Items for Systematic reviews and Meta-Analyses guidelines (PRISMA) (Supplementary Table 5). PubMed, Web of Science, MEDLINE, EMBASE, Google Scholar, and Cochrane Library were searched from January 1970 to June 2019 in order to identify relevant studies. We set the key word as “bilirubin” and (“chronic kidney disease” or “chronic renal disease” or “end-stage renal disease” or “end-stage kidney disease” or “estimated glomerular filtration rate”) without language limitation. The references in relevant reports, PubMed “related articles,” textbook chapters, and online clinical trial registries were searched to identify any related articles. Any unpublished data or incomplete data were requested by contacting authors through email.

### Selection Criteria

The inclusion criteria was as follows: (1) the studies investigated the relationship between bilirubin concentration and kidney disease progression, (2) cohort study or random clinical trials, (3) a comparison between highest STB group and lowest STB group or per unit STB increase, (4) followed participants for at least 12 months, (5) reported any of the following renal outcomes: progression to CKD or all-cause mortality, and (6) Relative risk (RR) with 95% confidence intervals CIs or the minimum information necessary to calculate these values were provided as effect size. Liver dysfunction which could lead to elevated levels of serum bilirubin were excluded in original research studies. Studies in review, editorials, letters, and case report forms were excluded from our meta-analysis. If the study did not meet the included criteria and could not provide categories of bilirubin or sufficient data to calculate effect size, then they were also excluded. The categorization for STB levels and the units used were in line with the definition in each study. In order to keep the unit of all included studies in accordance, μmol/L was converted to mg/dL divided 17.1. CKD was defined as a decline in estimated glomerular filtration rate (eGFR) <60 ml/min/1.73 m^2^ by using the CKD-EPI equation.

### Data Extraction and Quality Assessment

All included studies were independently identified by two investigators (JL and ZSL). Data extracted included age, sex, country of origin, study design, populations, serum bilirubin, follow-up, sample size, smoking status, body mass index, No. of cases, outcome, adjusted HR or RR or OR per unit of increase in baseline serum bilirubin, and those for highest and lowest group of STB levels. The studies which had several estimates adjusted HR for different numbers of potential confounders; the greatest number of potential confounding factors was selected for analysis. The quality of all included studies was assessed by two reviewers (JL and ZSL) using the Newcastle-Ottawa scale (NOS). The scores “7–9,” “4–6,” and “1–3” were considered as “high,” “moderate,” and “low” quality, respectively. Any discrepancies between the two investigators were discussed with a third independent reviewer (DWL).

### Outcomes

There were two outcomes included in this meta-analysis. The primary outcome was the assessment of kidney disease progression to CKD [defined as estimated glomerular filtration rate (eGFR) <60 ml/min/1.73 m^2^, doubling of serum creatinine, or 50% decline of kidney function or end-stage renal disease (ESRD)] and the secondary outcome was assessment of all-cause mortality in patients who developed CKD and underwent hemodialysis or peritoneal dialysis. The forest plots illustrate the two outcomes reproduced from the individual studies and the size of the symbol for the estimate is proportional to the weight of each study. A dashed vertical line and diamond at the bottom of the forest plot highlights the overall estimate and its 95% confidence interval.

### Statistical Analyses

In our study, the pooled RRs and 95% CIs were used for the association of STB with the risk of CKD and mortality. The hazard ratios (HR) and odds ratio (OR) in some original studies were assumed to provide accurate estimates of the risk ratio, so it was directly considered as RR (19). After certifying the connection between STB and kidney disease progression, we further clarified whether this link displays the dose–response effect and whether this dose–response relationship was nonlinear or linear. Detailed information is provided in the Supplementary Material. The Chi-square based *Q*-test and *I*^2^ were used to assess the heterogeneity among studies. Subgroup analysis and meta-regression were performed due to the potential impact of covariates on study heterogeneity (sample size, study design, study design, adjusted for albumin, adjusted for eGFR, adjusted for diabetes mellitus). Sensitivity analysis was also conducted by omitting each study one by one. Begg's test and Egger's test were used to evaluate publication bias. STATA and R software were used for statistical analyses and two-sided *P* < 0.05 was considered to be statistically significant.

## Results

### Literature Selection and Study Characteristics

**Figure 1:**
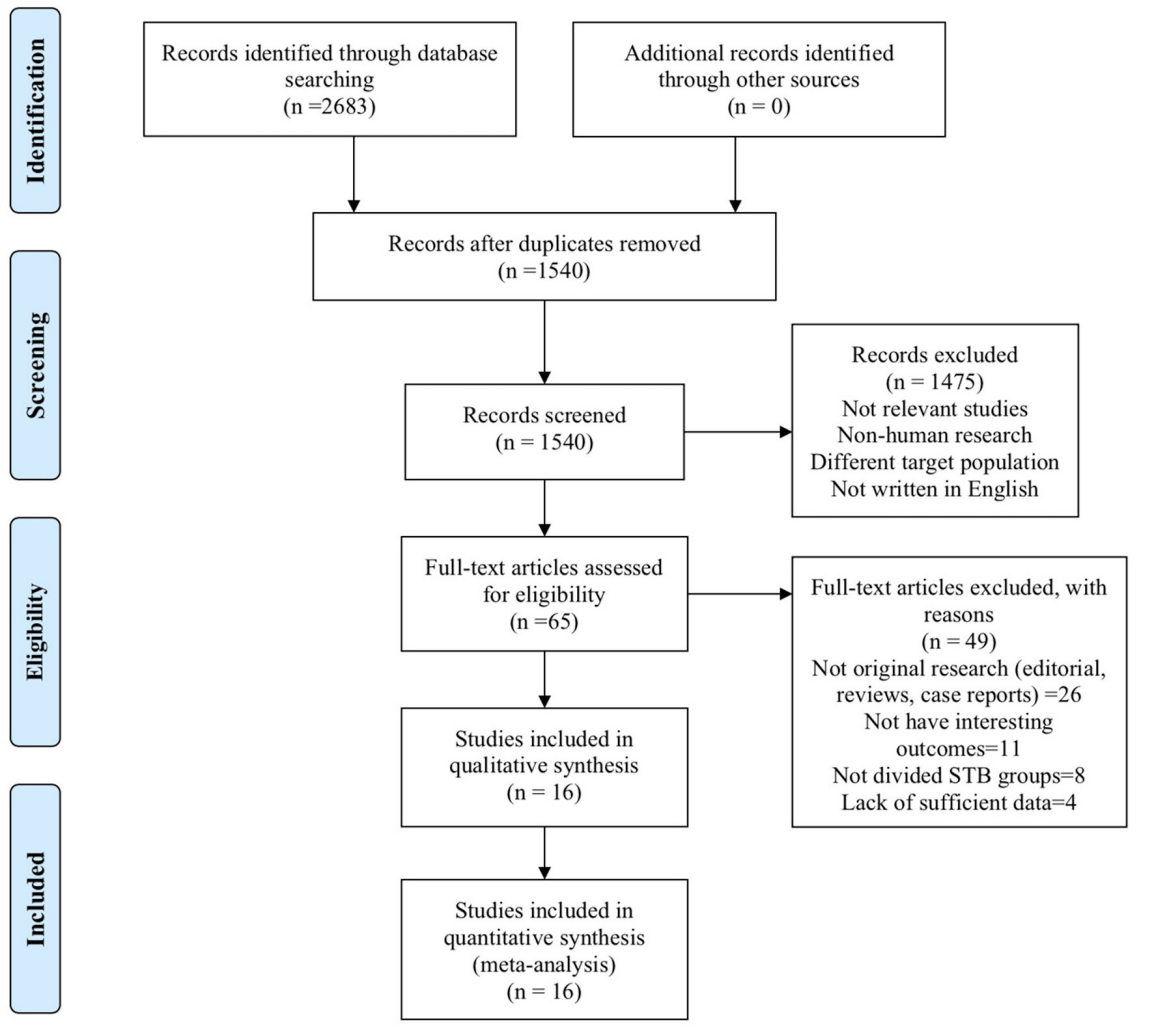
Flow chart of study selection.

A total of 2,683 studies were screened from the databases described above, and 16 publications (7–10, 15, 17, 18, 20–28) (19 data points) that met our criteria were included in the final selection (Figure 1). The characteristics of the included studies are provided in Table 1. The score of quality assessment for each study is shown in Supplementary Table 1. Two studies provided separate data for men and women. One study included two different trials (RENAAL and IDNT). Eleven studies (14 data points) provided a comparison between highest and lowest STB levels, and seven studies (10 data points) were viable for analysis on the effect of per 0.1 mg/dl STB level increase. Further details on categories and continuous STB levels are shown in Supplementary Table 2.

**Table 1:** Characteristics of included studies in this meta-analysis (*n* = 16).

**Author**	**Year**	**Country**	**Populations**	**Cohort designation**	**No. of participants**	**Age (year)**	**Male (%)**	**Follow-up**
Chin	2009	Korea	IgA nephropathy	CS	1,469	36.3	56.11%	44.9 months
Kawamoto	2014	Japan	Elderly adults	CSS	1,050	80.21	39.30%	3 years
Tanaka M (men)	2014	Japan	General population	CS	1,627	47.7 ± 9.7	100.00%	7.7 years
Tanaka M (women)	2014			CS	1,157	46.3 ± 9.7	0.00%	7.7 years
Ryu	2014	Korea	General population	CS	12,823	37.2 ± 4.9	100%	7 years
Riphagen (RENAAL)	2014	Netherland	Diabetes nephropathy	RCT	1,498	60.1 ± 7.4	63.20%	3.4 years
Riphagen (IDNT)	2014			RCT	1,707	58.9 ± 7.8	66.40%	2.6 years
Tanaka S	2015	Japan	IgA nephropathy	RCS	694	36	47.42%	4.9 years
Sakoh	2015	Japan	CKD	PCS	279	73	69.00%	21 months
Lee (men)	2015	Taiwan	General population	CSS	2,260	51.8 ± 12.2	100.00%	NA
Lee (women)	2015			CSS	1,616	49.3 ± 11.8	0%	NA
Wang	2016	China	Type 2 diabetes patients	PCS	2,958	64.06	46.55%	5 years
Ahn Hee	2017	Korea	Type 2 diabetes patients	RCS	349	55 ± 11.7	39%	41 months
Su	2017	Taiwan	Hemodialysis patients	RCS	47,650	61.4 ± 13.6	50%	3 years
Yang	2017	Taiwan	Peritoneal dialysis patients	RCS	3,704	53.5 ± 15.0	44%	2.12 ± 1.07 years
Liu	2018	China	CKD	RCS	316	61.7 ± 10.9	56%	29.09 months
Wang	2018	China	Hypertension adults	RCT	12,633	59.59	37.78%	4.4 years
Wu	2019	China	Diabetic nephropathy	RCS	118	52.58 ± 9.36	67.80%	25 months
Tsujikawa	2019	Japan	Peritoneal dialysis patients	PCS	94	55.5 ± 14.2	66.00%	3 years

*CS, cohort study; CSS, cross-sectional study RCT, randomized controlled trials; PCS, prospective cohort study; RCS, retrospective cohort study; RENAAL, The Reduction in End Points in NIDDM with the Angiotensin II Antagonist Losartan study; IDNT, the Irbesartan Diabetic Nephropathy Trial; CKD, Chronic Kidney Disease; NA, not applicable*.

### Association of Serum Bilirubin With the Risk of CKD

**Figure 2:**
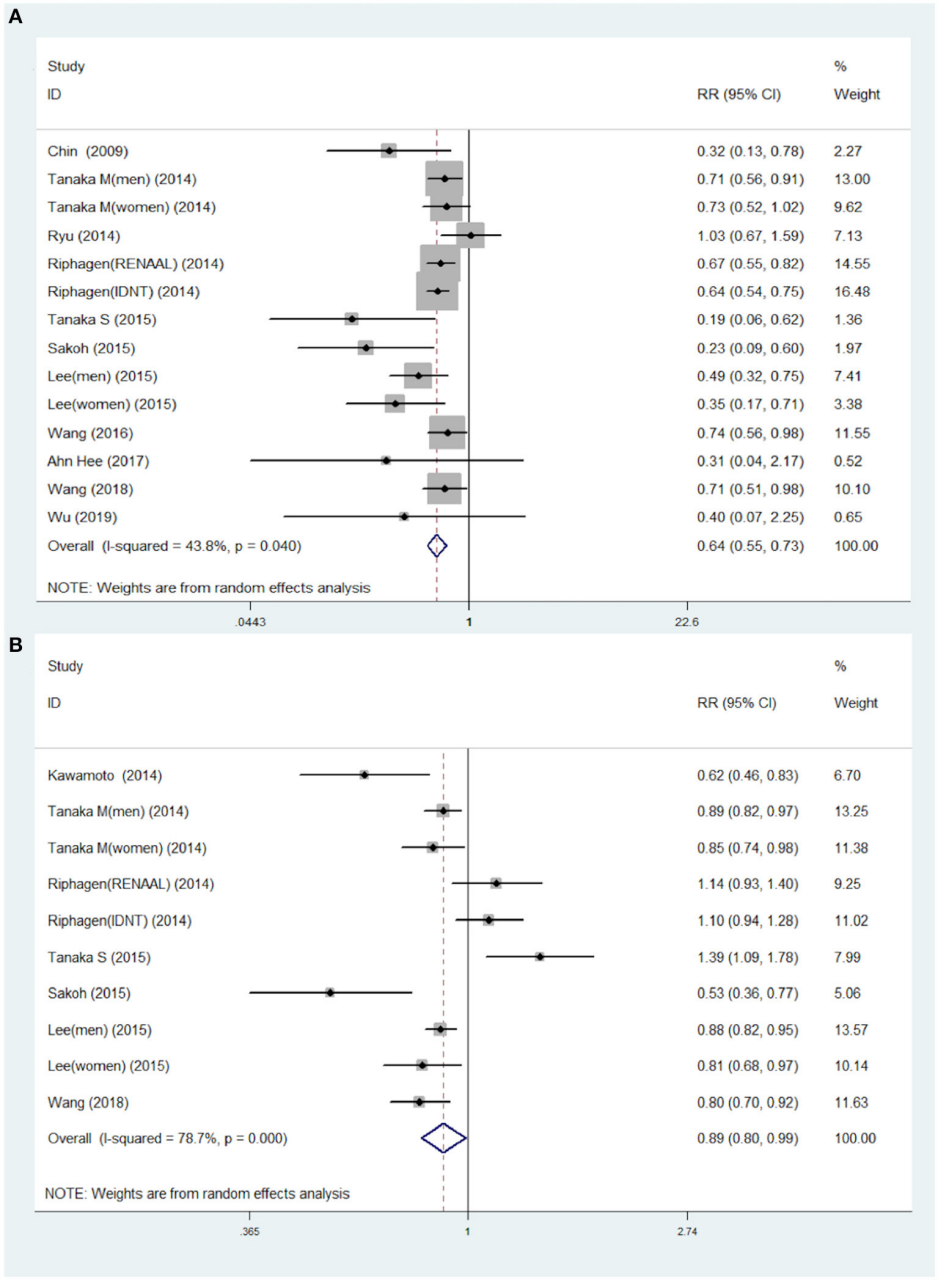
Pooled RR with 95% CIs for the association of serum total bilirubin levels and the risk of CKD for the highest compared to the lowest category group **(A)** and for each 0.2 mg/dl increase **(B)**. The random-effects model was used and the area of each square stands for the weight of each study in the meta-analysis. The square sizes are proportional to the weight of each study in the meta-analysis; the diamond shows the overall RR; the horizontal lines indicate the 95% confidence intervals (CIs). CKD, chronic kidney diseases; RR, risk ratio.

The association of STB levels with risk of kidney disease progression were analyzed by the RR of the highest STB levels compared to the lowest STB levels in 11 studies (14 data points). The results highlighted that patients with the highest STB levels were associated with lower risk of CKD progression (RR = 0.64; 95% CI 0.55–0.73), as shown in Figure 2A. This tendency was also consistent with the results of per 0.2 mg/dl increases in STB levels. Although moderate heterogeneity among the 11 studies (*I*^2^ = 43.8%, *P* = 0.04) was found, this also demonstrates the association between the STB and the progression to CKD. The RR for per 0.1 mg/dl increases in serum bilirubin were extracted from seven studies that included 10 data points, and were used for continuous variable analysis. Considering clinical experience, each 0.1 ml/dl increase is of little significance, so we calculated the risk ratio (RR) for 0.2 mg/dl increases (29). To elaborate, each 0.2 mg/dl serum bilirubin increase was associated with an 11% decreased risk for progression to CKD. The pooled RR and 95% CI were 0.89, 95% CI (0.80–0.99), as shown in Figure 2B (*I*^2^ = 78.7%; *P* < 0.0001).

### Association of Serum Bilirubin With the Risk of Mortality

All-cause mortality was extracted from only four studies, including 51,764 patients. The adjusted HR of all-cause mortality for highest STB levels compared to the lowest STB levels was 0.77 (95% CI 0.42–1.41) in dialysis patients with heterogeneity at 84% (Supplementary Figure 1). Two of the studies proved that higher serum bilirubin was linked to a higher risk of mortality, while the other two studies had contrary conclusions. Our results illustrated that an increase of STB did not have a significant association with the risk for mortality in dialysis patients.

### Publication Bias and Sensitivity Analyses

Begg's and Egger's test were performed for 11 studies on association of STB levels with disease progression. The results show that publication bias did exist, and trim and fill methods were adapted to adjust for the publication bias (Supplementary Figures 2, 3). Sensitivity analyses were conducted to assess the extent of the influence of single studies on the pooled RR. The results indicated that there was no single study that dramatically influenced the pooled RR (Supplementary Figure 4). Detailed procedures and results are included in the Supplementary Material.

### Subgroup and Regression Analyses

**Figure 3:**
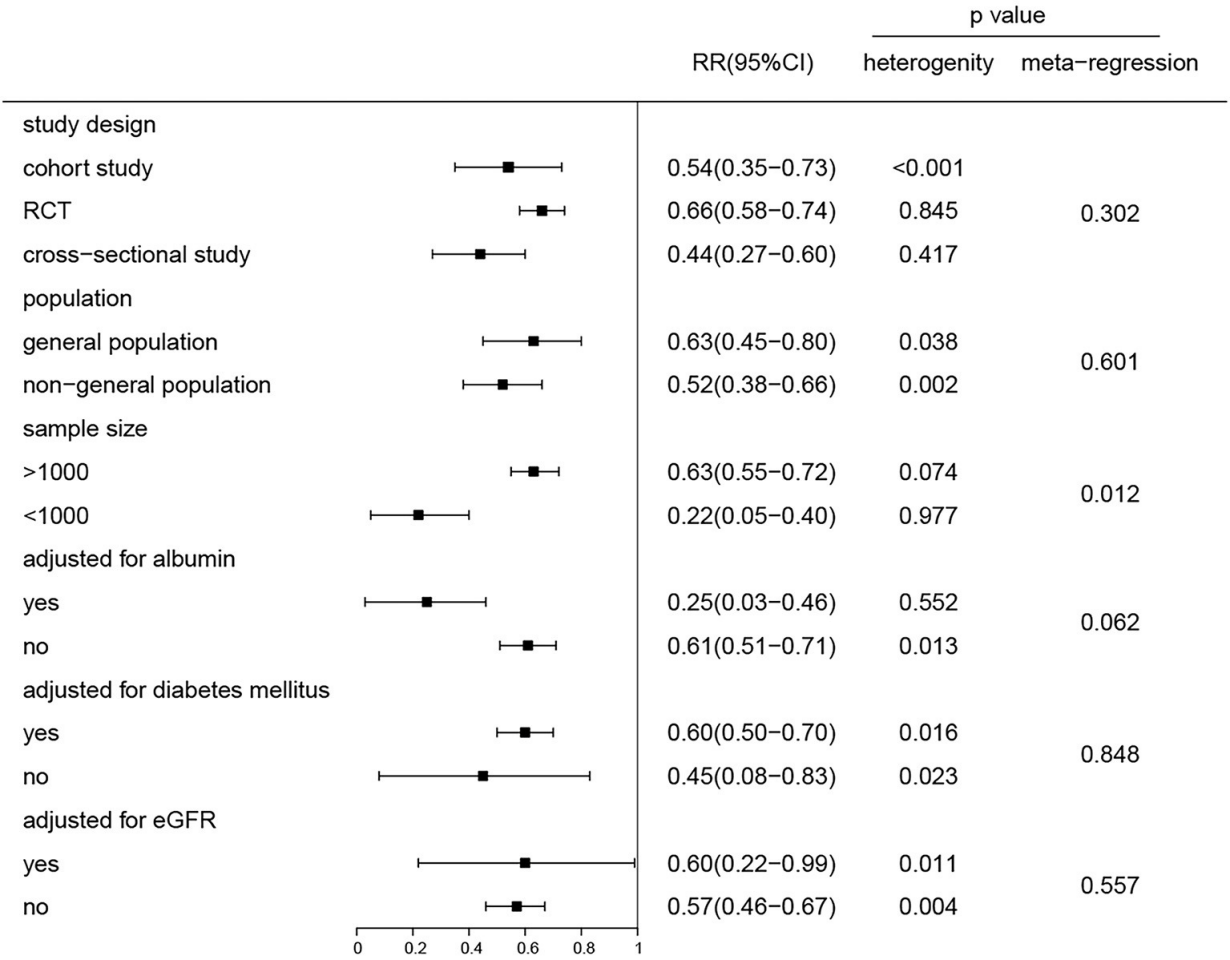
Subgroup and regression analyses of the association between the serum total bilirubin levels and the risk of CKD for the highest compared and the lowest category group. P for the heterogeneity within each subgroup.

We analyzed the effect of potential impacts of covariates on study heterogeneity through stratified analyses and meta-regression. The gradually elevated STB levels with decreased risk of CKD remained for all factors (all RR were <1) (Figure 3). The negative association between the STB levels and the CKD risk was found to be significant in both the general population and nephropathy patients. Adjustment for albumin, diabetes mellitus, and eGFR were not found to have an influence on the relationship between the STB levels and risk of CKD (*P* = 0.06, 0.84, 0.55, respectively). Although there were significant differences in sample size >1,000 and <1,000 of the groups, the association between the STB levels and risk of CKD were both significant. We also conducted a regression analysis to evaluate the reason for the association between STB levels and all-cause mortality. There were no significant differences in the subgroup adjustment for albumin, diabetes mellitus, or eGFR except for sample size (*P* = 0.04) (Supplementary Figure 5).

### Linear Dose–Response Analyses

**Figure 4:**
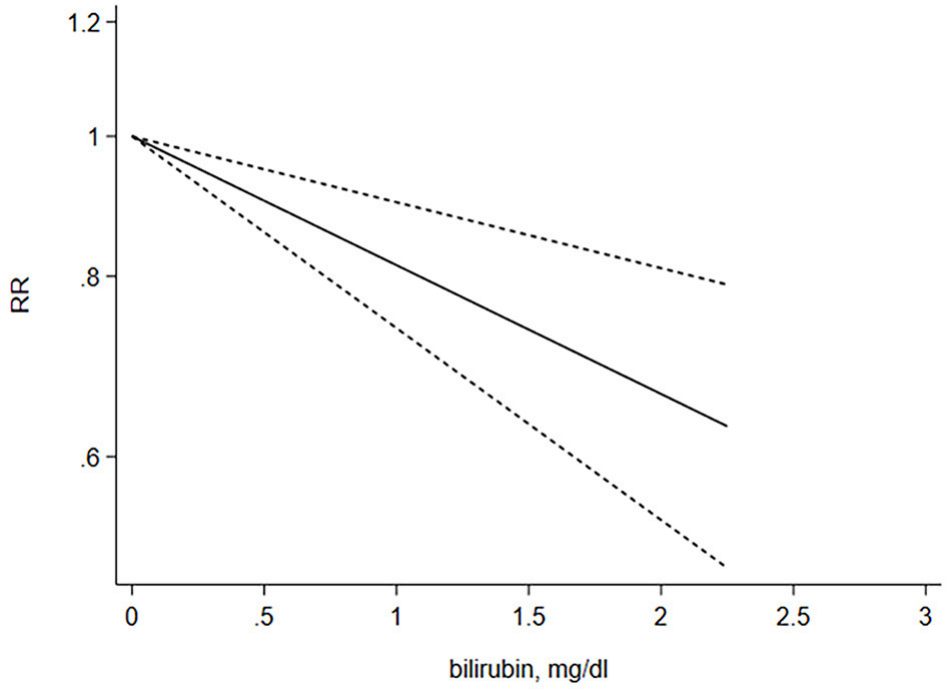
Dose–response relationship between the serum total bilirubin levels and risk of CKD. The liner dose–response model is based on restricted cubic splines for STB concentrations at three point (25, 50, 75 percentiles). The line with short dashes represents the 95% confidence intervals.

A restricted cubic spline regression model was used to explore potential linearity. A total of seven studies that provided sufficient data were used to analyze the relationship between STB levels and risk of CKD. A linear dose–response relationship was illustrated using random-effects analysis and the results showcased a prominent, negative linear relationship (X^2^ = 14.70; *P* < 0.001). The overall RR per 1 mg/dl increasement was associated with a 20% reduction in risk of CKD (95% CI 0.72–0.90), *P* < 0.001). No significant heterogeneity among the included studies was found (Q = 15.13, *P* = 0.36) (Figure 4).

## Discussion

Recently, many studies have revealed that STB levels play a critical role in CKD progression and mortality, but other studies have had inconsistent conclusions. Whether or not lower STB levels could be a biomarker of reduced kidney function is currently unclear. As far as we know, this is the first meta-analysis to focus on both CKD progression and all-cause mortality. Our meta-analysis demonstrates that higher STB levels may serve as protective factors for the development of CKD and elevated STB levels could lead to 36% of a significant decrease in the risk of CKD progression. However, the meta-analysis also concluded that there was no significant relationship between STB levels and risk of all-cause mortality.

Serum bilirubin has long been recognized as an abnormal sign of liver dysfunction. Recent data strongly indicates that slightly increased STB concentration can be a potent biological protective marker. In humans, low (<7 mmol/L) STB levels may be a risk factor for systemic diseases associated with increased oxidative stress, such as cardiovascular diseases (CVD) (30), diabetes (31), metabolic syndrome (32), certain cancers (33), and autoimmune diseases (34). However, the relationship between STB levels and kidney disease outcomes and mortality have been less certain. Based on 11 studies and 41,188 participants, our meta-analysis displayed a pooled analysis (RR = 0.64 95% CI 0.55–0.73) of the highest compared to the lowest STB group and demonstrated that STB levels were associated with CKD progression. Clinically speaking, each 0.1 ml/dL increase is of little significance, and therefore, we calculated the RR for 0.2 mg/dl, upon which the results showed a stronger association between the STB levels and renal outcomes (RR = 0.89, 95% CI 0.80–0.99). Meanwhile, a dose–response analysis showed that a 1 mg/dl increment in the STB levels led to a 20% decrease in the risk of CKD, regardless of various study characteristics. This result was consistent with a linear dose–response relationship. To assess the consistency of the association between STB levels and development of CKD, we conducted subgroup and meta-regression analysis stratified by potential confounders. STB is mainly transported in combination with albumin in the blood and a small amount is combined with α1 globulin for complex transport. Targher et al. (35) demonstrated that STB was inversely associated with eGFR in the general population in the US while Shin's et al. (36) research has concluded the opposite. Hence, according to whether the STB levels adjust for albumin and eGFR, subgroup analyses were performed and the results indicated that the significant relationship between STB and the development of CKD was not affected by stratified factors, except for sample size. When the sample size is smaller than 1,000, it has a higher heterogeneity. This could be explained by the fact that a small sample size has a different definition for the quartile of the STB levels.

Bilirubin, as the product of heme, has long been considered a symbol of liver dysfunction or a potentially harmful element that causes neonatal jaundice. Recent evidence has shown that mildly elevated bilirubin levels within the physiological range have shown to be protective against various diseases. Our meta-analysis identified higher STB levels that were validated for predicting survival of incident CKD. The potential mechanisms behind the protective role of serum bilirubin are as follows: First, serum bilirubin is seen as a potent, endogenous antioxidant reagent because of persistent recovery in intracellular bilirubin redox metabolism. Second, it has been previously demonstrated that STB has a positive association with anti-inflammatory effects, which are a protective factor of CKD (37). Zucker et al. supported the finding that bilirubin could prevent an inflammatory reaction in a mice model of inflammatory colitis by preventing vascular cell adhesion molecule 1 (VCAM-1) -mediated leukocyte infiltration in target tissues (38). Third, renal endothelial dysfunction plays a critical role in progression of CKD and renal fibrosis through the process of endothelial-to-mesenchymal transition (End MT) (39). Higher STB levels are associated with lower levels of oxidative stress and enhancement of endothelium-dependent vasodilation in Gilbert's syndrome patients (40). Vogel M.E reported that bilirubin inhibits monocyte migration across activated human endothelial cells by disrupting the VCAM-1/ICAM-1 signaling pathway but does not affect the expression of the two *in vitro* or *in vivo* (41). Finally, diabetic nephropathy is the most common cause of ESRD, which accounts for >40% of patients on renal replacement therapy (42). One study depicts that in diabetic db/db mice, hyperbilirubinemia has a protective effect against the mesangial expansion and progression of CKD (43). Moreover, biliverdin and conjugated bilirubin may have an anticomplement role (44). More specifically, the protective effects of STB are complex and include multiple stages of cell and tissue biology. These biological properties of bilirubin support the finding that it plays a protective role for STB levels associated with the renal outcomes.

Patients with ESRD often undergo renal replacement therapy, including hemodialysis and peritoneal dialysis. The dialysis removes water-soluble circulating antioxidants, including uric acid and ascorbate, but does not remove hydrophobic substances, such as unconjugated bilirubin, which is plasma albumin-bound (18). There is a need to find appropriate biomarkers that indicate the clinical outcomes of dialysis patients. Therefore, we analyzed the relationship between STB levels and mortality in ESRD patients who underwent dialysis. The results showed no significant improvement in mortality outcomes within this dialysis population. There may be a few reasons behind this. First, only four studies and 51,764 participants were included in our meta-analysis, which led to high heterogeneity and publication bias. The way of dialysis, study design, adjusted items, and precise detecting time of STB could have influenced the negative outcomes. Second, Liu and Tsujikawa's studies have 316 and 94 patients, respectively. This could have caused low quality of the two studies. Third, in patients with ESRD, muscle wasting is accelerated by several catabolic factors. Higher bilirubin levels are often associated with lower triglycerides and cholesterol levels, low testosterone levels (45), and abnormalities in the insulin growth factor-1 pathway (46). Vitek et al. demonstrated that there was a strong negative association between bilirubin levels and total mortality in the general population, especially in men. There are significant differences between dialysis and non-dialysis patients in regards to metabolic syndrome (47). It is postulated that patients with dialysis have higher mortality rates possibly because of the potent confounding effect of malnutrition and inflammation in hemodialysis patients with a low BMI index. Mortality rates for maintenance dialysis patients are much higher than the general population and this may preclude identification of small effect size risk factors. Thus, clinical trials and further research into this matter are needed to evaluate whether higher STB levels can reduce mortality rates in dialysis patients.

This study did have several strengths. Specifically speaking, this is the first meta-analysis of studies that examined associations of STB levels for renal outcomes among different subgroup populations and also examined associations between STB and mortality in dialysis populations. Furthermore, we not only conducted the highest STB groups verse reference groups analyses but also continuous variables (per 0.2 mg/dl increase) were used to certify the dose–response relationship.

Nonetheless, there are potential limitations to this study. First, our meta-analysis was restricted to published aggregate data. Individual participant-level data were not available, which is the primary limitation of this meta-analysis. Unpublished data or incomplete retrieval of identified studies led to an incomplete set of evidence and produced biased effects in the summary results. Funnel plot and “trim and fill” methods were used for assessment to balance out the publication bias and the relationship between the STB levels, and the risk of CKD remained statistically significant. Second, the included studies do not directly report on the data information required for meta-analysis, which can cause reporting bias. Third, potential confounding factors, such as age, sex, history of smoking, and/or alcohol intake (all of which could potentially influence the STB levels or risk of disease progression to CKD), could not be excluded. The studies did not adjust for same risk factors. Considering this fact, we included these factors in the multiple adjust models as much as possible. Fourth, only a few studies have differentiated the conjugated bilirubin and unconjugated bilirubin, and therefore the accurate role of separating bilirubin could not be investigated. Fifth, only STB levels in a narrow range (1.5 times the upper limit of normal) were studied. To translate these findings to clinical practice, future studies are needed to define an optimal range of STB. Finally, there was only one study involving a European population, which makes it difficult to definitively assess the association between STB levels and CKD in American and African populations.

In conclusion, our study indicates that individuals with reduced bilirubin concentrations, in the absence of liver pathology, are at a higher risk of CKD. This association was confirmed to be a linear dose–response relationship. Whether high STB levels serve as a protective factor of mortality or the risk of renal replacement therapy among the patients that underwent dialysis remains inconclusive. High-quality randomized controlled trials are needed to target STB levels for predicting renal outcomes. Considering that multiple mechanisms likely explain the protective properties of bilirubin and that bilirubin measurement is performed routinely for most patients, bilirubin might be a potential predictor for renal prognosis. It also highlights the importance of monitoring biomarkers related to serum bilirubin homeostasis in early prevention, diagnosis, and treatment of CKD and provides evidence to further multicenter validation research for bilirubin levels in routine risk stratification of CKD.

## Author Contributions

JL: conceptualization, formal analysis, methodology, software, and writing—original draft. JL, DL, and ZL: data curation. DL and ZL: investigation and supervision. ZL: project administration, validation, and writing—review and editing. All authors contributed to the article and approved the submitted version.

## Conflict of Interest

The authors declare that the research was conducted in the absence of any commercial or financial relationships that could be construed as a potential conflict of interest.
