# Supplementary material for: Serum Total Bilirubin and Progression of Chronic Kidney Disease and Mortality: A Systematic Review and Meta-Analysis
Source: Front Med (Lausanne). 2021 Jan 25;7:549. doi: 10.3389/fmed.2020.00549 (PMC7868400; doi:10.3389/fmed.2020.00549)
Supplement: Supplementary file 1 [file Data_Sheet_1.docx]

**Supplement materials**

Supplemental Methods: Statistical analyses; Publication bias and sensitivity analyses.

Supplemental Table S1. Quality assessment of included studies.

Supplemental Table S2. Summary of serum total bilirubin levels in the meta-analysis.

Supplemental Figure S1. Association of serum bilirubin with the risk of mortality.

Supplemental Figure S2. Publication bias for 11 studies about the association of STB levels with the highest compared to the lowest group.

Supplemental Figure S3. Publication bias for 7 studies about the effect of per 0.2mg/dl serum total bilirubin levels.

Supplemental Figure S4. Sensitivity analyses about the influence of single studies on the pooled RR.

Supplemental Figure S5. Subgroup and regression analyses of the serum total bilirubin levels and the mortality.

**Supplemental methods**

**Statistical analyses**

Random-effects models were applied for the incorporation of between-study heterogeneity and to obtain an overall RR for the categorical variable (highest STB levels category compared with the lowest STB levels category) and continuous variable (per unit STB increase). The RR of category STB levels increment for each study and restricted cubic spline regression model with three points (25,50,75percentiles) and generalized least squares estimation (GIST) method was used to estimate the change in exposure effect for each dose effect. We first according to the nonlinearity model goodness of fit test results to determine whether the model needs to consider the heterogeneity of trends between different studies. If the *P* value for heterogeneity of the previous model was<0.05, a random-effects potential nonlinearity model. The model independent variable consists of three regression splines. If the *P* value of regression coefficient of the regression spline is <0.05, it is a nonlinear dose response relationship. Otherwise, change to a linear regression model.

**Publication bias and sensitivity analyses**

The results show that publication bias was exist in Egger’s test (*P*=0.028) (Supplemental Figure S2A) but not in Begg’s test (*P*=0.101) (Supplemental Figure S2B). Therefore, trim-and fill methods were adapted to adjust the publication bias and 4 hypothetical positive unpublished studies were added. After modified the association between the STB concentration and the risk of CKD was slightly changed (RR=0.67; 95%CI, 0.57-0.79), but still have the statistical significance (*P*< 0.0001) (Supplemental Figure S2C). Begg’s and egger’s test for 7 studies about the effect of per 0.2mg/dl STB levels increase were also analysis and the results show in Supplemental Figure S3. Sensitivity analyses were conducted to assess the extent of the influence of single studies on the pooled RR. The results indicate no single study dramatically influenced the pooled RR. The pooled RRs range from 0.62 (95%CI, 0.52-0.74) to 0.65 (95%CI, 0.56-0.75) comparing the highest to the lowest group in the STB levels. Furthermore, analysis using per 0.2mg/dL STB increment suggested that there is no evidently publication bias and omitting individual study has no influenced the results (Supplemental Figure S4).

**Supplemental Table S1.** Quality assessment of included studies on serum total bilirubin and risk of chronic kidney disease and mortality.

| study | year | | | selection | comparability | outcome/exposure | overall quality |
| --- | --- | --- | --- | --- | --- | --- | --- |
| Chin | | 2009 | 4 | | 2 | 1 | 7 |
| Kawamoto | | 2014 | 2 | | 2 | 2 | 6 |
| Tanaka M | | 2014 | 3 | | 1 | 3 | 7 |
| Ryu | | 2014 | 4 | | 2 | 2 | 8 |
| Riphagen | | 2014 | 3 | | 2 | 3 | 8 |
| Tanaka S | | 2015 | 2 | | 1 | 3 | 6 |
| Sakoh | | 2015 | 4 | | 1 | 2 | 7 |
| Lee | | 2015 | 3 | | 1 | 2 | 6 |
| Wang | | 2016 | 4 | | 1 | 3 | 8 |
| Ahn Hee | | 2017 | 2 | | 1 | 3 | 6 |
| Su | | 2017 | 4 | | 2 | 2 | 8 |
| Yang | | 2017 | 3 | | 1 | 3 | 7 |
| Liu | | 2018 | 2 | | 1 | 2 | 5 |
| Wang | | 2018 | 3 | | 2 | 3 | 7 |
| Wu | | 2019 | 2 | | 2 | 2 | 6 |
| Tsujikawa | | 2019 | 2 | | 2 | 2 | 6 |

**Supplemental Table S2.** Summary of serum total bilirubin levels in the meta-analysis.

| Author | Year | outcome | bilirubin | OR/RR/HR | adjust items |
| --- | --- | --- | --- | --- | --- |
| Chin | 2009 | CKD5 | Q1(<0.4 mg/dL) | 1.00 | age, gender, hypertension, systolic blood pressure, diastolic blood pressure, serum albumin, serum creatinine, urine protein 3+ or more by dipstick test, and bilirubin group |
|  |  |  | Q4 (>0.8 mg/dL) | 0.32 (0.13–0.77) |  |
| Kawamoto | 2014 | CKD5 | per 0.2mg/dl increase | 0.62 (0.462-0.83) | gender, age, body mass index, smoking status, diastolic blood pressure, antihypertensive medication, triglycerides, high density lipoprotein cholesterol, low density lipoprotein cholesterol, anti dyslipidemic medication, fasting blood glucose, antidiabetic medication, and uric acid |
| Tanaka M(men) | 2014 | CKD | lowest group (<0.6 mg/dL) | 1.00 | age, history of smoking or alcohol intake, BMI, systolic blood pressure (SBP), FPG, LDL，cholesterol, triglycerides, GGT, AST, ALT, UA, creatinine, total leukocyte count, total bilirubin and medication for hypertension, diabetes or dyslipidemia |
|  |  |  | highest group (>0.8 mg/dL) | 0.71(0.56-0.91) |  |
|  |  |  | per 0.05mg/dl increase | 0.97(0.95-0.99) |  |
| Tanaka M(women) | 2014 | CKD | lowest group (<0.5 mg/dL) | 1.00 |  |
|  |  |  | highest group (>0.7 mg/dL) | 0.73(0.52-1.02) |  |
|  |  |  | per 0.05mg/dl increase | 0.96(0.93-1) |  |
| Ryu | 2014 | CKD | Q1<0.9 mg/dL | 1.00 | age, smoking, alcohol intake, exercise GFR, CRP, AST, ALT, GGT and metabolic syndrome traits (high glucose, high blood pressure, low HDL cholesterol, high triglycerides and high BMI |
|  |  |  | Q4>1.5 mg/dL | 1.03（0.67-1.59） |  |
| Riphagen (RENAAL) | 2014 | CKD5 | total group (0.57±0.19) | 0.67（0.55-0.83） | adjusted for age, sex, baseline eGFR, baseline log ACR, race, smoking, history of CVD, baseline BMI, total cholesterol, diastolic blood pressure, HbA1c, treatment assignment, and log AST |
|  |  |  | per 0.1mg/dl increase | 1.07(0.97-1.19) |  |
| Riphagen (IDNT) | 2014 | CKD5 | total group (0.54±0.21) | 0.64（0.55-0.76） |  |
|  |  |  | per 0.1mg/dl increase | 1.05(0.97-1.13) |  |
| Tanaka S | 2015 | CKD5 | Q1<0.40 mg/dL | 1.00 | age, sex and clinical parameters (serum albumin, urinary protein excretion, estimated glomerular filtration rate) and pathological parameters (mesangial hypercellularity score, segmental glomerulosclerosis, tubular atrophy/interstitial fibrosis) |
|  |  |  | Q5 >0.80 mg/dL | 0.19(0.06-0.63) |  |
|  |  |  | per 0.1mg/dl increase | 1.18(1.04-1.33) |  |
| Sakoh | 2015 | CKD5 | lowest group ≤0.3 mg/dL | 1.00 | adjusted for age and sex, smoking, diabetes mellitus, systolic blood pressure, and dyslipidemia, use of immunosuppressants, use of RAAS inhibitors, daily proteinuria, hemoglobin, eGFR, and serum phosphorus. |
|  |  |  | highest group >0.8 mg/dL | 0.23(0.08-0.55) |  |
|  |  |  | per 0.1mg/dl increase | 0.73(0.6-0.87) |  |
| Lee(men) | 2015 | CKD | Q1 <0.5 mg/dL | 1.00 | age, BMI, smoking, alcohol use, hypercholesterolemia, hyper- triglyceridemic, low HDL-C, use of anti-hyperlip- idemic medications, hypertension, diabetes, hyperuricemia, and history of CVD |
|  |  |  | Q4 >0.9 mg/dL | 0.49 (0.32-0.74) |  |
|  |  |  | per 0.1mg/dl increase | 0.94(0.91-0.98) |  |
| Lee(women) | 2015 | CKD | Q1 <0.4 mg/dL | 1.00 |  |
|  |  |  | Q4 >0.8 mg/dL | 0.35 (0.17-0.70) |  |
|  |  |  | per 0.1mg/dl increase | 0.9(0.82-0.98) |  |
| Wang | 2016 | CKD | Q1<0.70 mg/dL | 1.00 | age, sex, central obesity, education, smoking status, drinking status, physical activity, systolic pressure, triglyceride, high density lipoproteins, use of medications (hypotensor, lipid-lowering)，liver function (alkaline phosphatase, alanine aminotransferase, aspartate aminotransferase)，fasting plasma glucose, use of antidiabetic, and duration of diabetes， use of aspirin |
|  |  |  | Q3 >0.90 mg/dL | 0.74 (0.56-0.98) |  |
| Ahn Hee | 2017 | CKD | Q1 <0.55 mg/dL | 1.00 | NA |
|  |  |  | Q4 >0.88 mg/dL | 0.31(0.04-1.96) |  |
| Su | 2017 | Death | Q1<0.3 mg/dL | 1.00 | age, sex，ALT, ferritin, uric acid, albumin, Hb, Ca, P, Kt/V, WBC, BUN, Cr, residual GFR |
|  |  |  | Q4>0.7 mg/dL | 1.24（1.00-1.55） |  |
|  |  |  | per 0.1mg/dl increase | 1.05（1.01-1.09） |  |
| Yang | 2017 | RRT/Death | middle group 0.5-0.6mg/dL | 1.00 | age, sex, diabetes mellitus, hypertension, heart failure, left ventricular hypertrophy, cerebrovascular accident, coronary artery disease, history of myocardial infarction, and antihypertensive agents, aspartate aminotransferase, alanine aminotransferase, ferritin, iron, uric acid, total iron binding capacity, albumin, hemotocrit, calcium, phosphorus, intact-parathyroid hormone, alkaline phosphatase, Kt/V (a measure of dialysis adequacy) |
|  |  |  | highest group >0.6mg/dL | 1.44(1.08-1.92) |  |
| Liu | 2018 | RRT/Death | Q1 <0.29 mg/dL | 1.00 | sex, history of smoking, hemoglobin, serum albumin, alanine aminotransferase, aspartate aminotransferase, serum phosphorus, calcium, 24-hour urine protein |
|  |  |  | Q4 >0.66 mg/dL | 0.12 (0.03–0.38) |  |
| Wang | 2018 | CKD5 | T1<0.50 mg/dL | 1.00 | age, sex, treatment group, systolic blood pressure, body mass index, smoking status, alcohol intake, eGFR, proteinuria, serum glucose, total cholesterol, alanine aminotransferase, aspartate transaminase at baseline, and time-averaged systolic blood pressure during treatment |
|  |  |  | T3 >0.68 mg/dL | 0.71（0.51-0.97） |  |
|  |  |  | per SD increase | 0.8(0.7-0.92) |  |
| Wu | 2019 | CKD5 | low group <0.43 mg/dL | 1.00 | gender, age, the duration of diabetes, hypertension, anemia, serum glucose and eGFR |
|  |  |  | high group>0.43mg/dL | 0.40(0.07-2.22) |  |
| Tsujikawa | 2019 | RRT | T1 <0.30 mg/dL | 1.00 | age, sex, diabetic nephropathy, renal Kt/V, and hemoglobin |
|  |  |  | T3 >0.30mg/dL | 0.26(0.06-1.0) |  |
|  |  |  | per 0.1mg/dl increase | 1.50(1.01-2.51) |  |

**Supplemental**
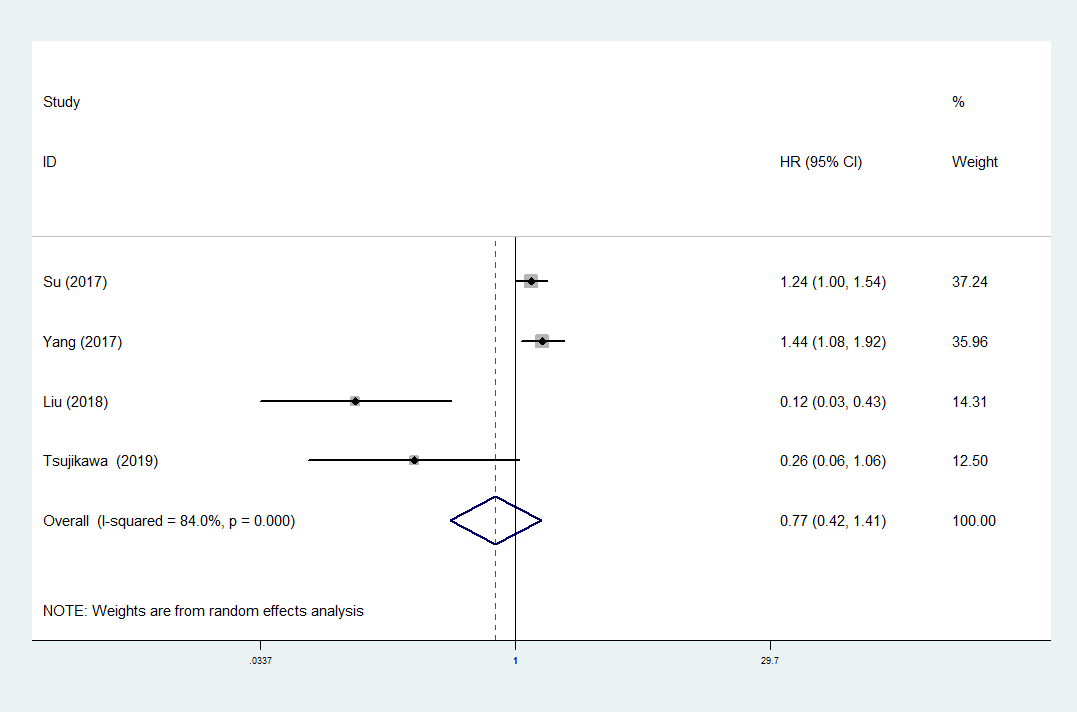
**figure S1.** Association of serum bilirubin with the risk of mortality.

Random-effects model analysis for overall HR (0.77, 95% CI=0.42-1.41) of mortality for the highest compared with the lowest category of serum total bilirubin levels.

**Supplemental figure S2.** Publication bias for 11 studies about the association of STB levels with the highest compared to the lowest group.


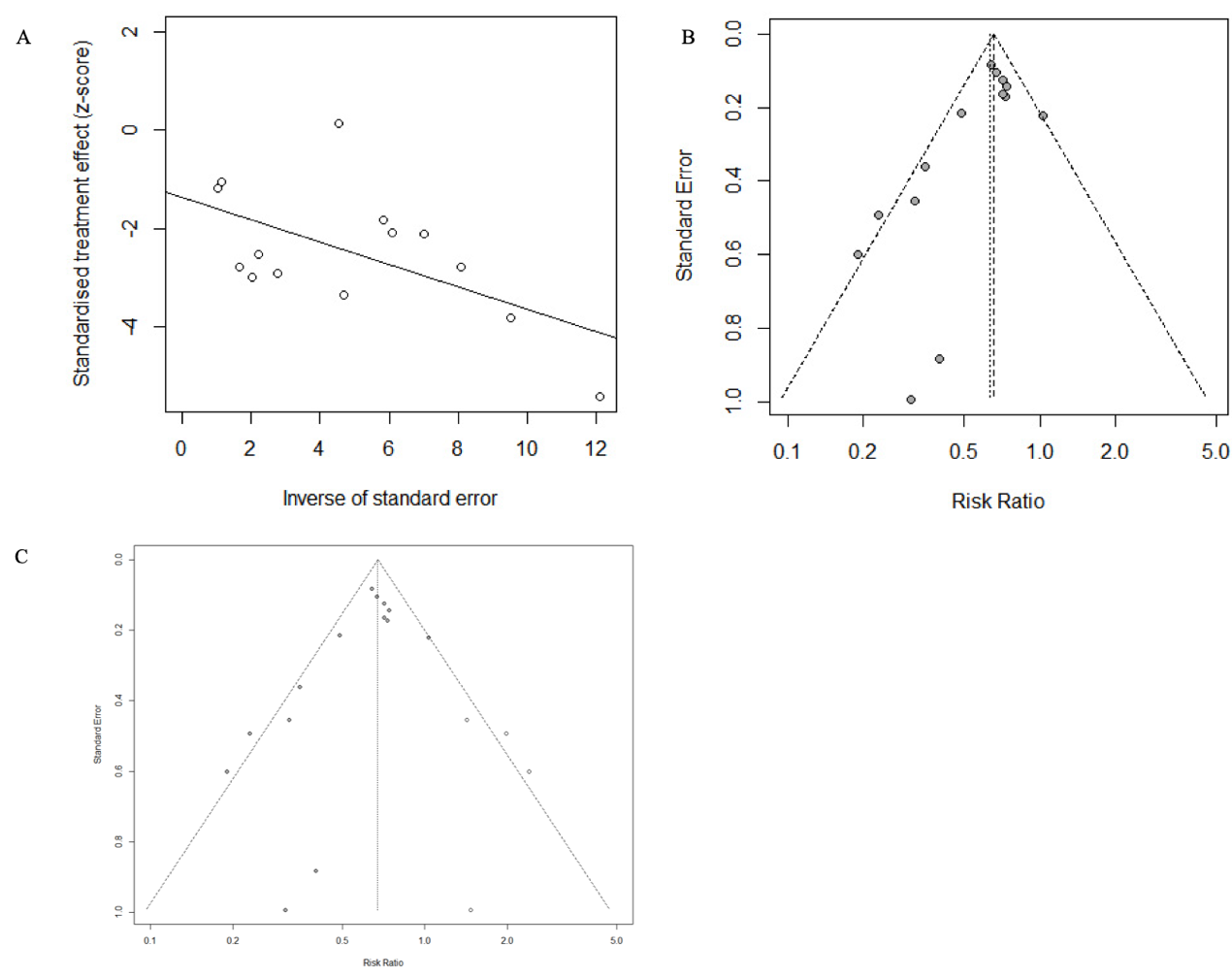


1. Funnel plot with Egger’s test for the publication bias; (B) Begg’s funnel plot analysis to assess publication bias: Each point represents a separate study included in this meta-analysis; (C) Egger’s funnel plot after “trim-and-fill” procedure to adjust publication bias: Solid circles means study included in this meta-analysis; Hollow circles means four hypothetical unpublished prospective studies that distorted the symmetry of the funnel plot.

**Supplemental figure S3.** Publication bias for 7 studies about the effect of per 0.2mg/dl serum total bilirubin levels.


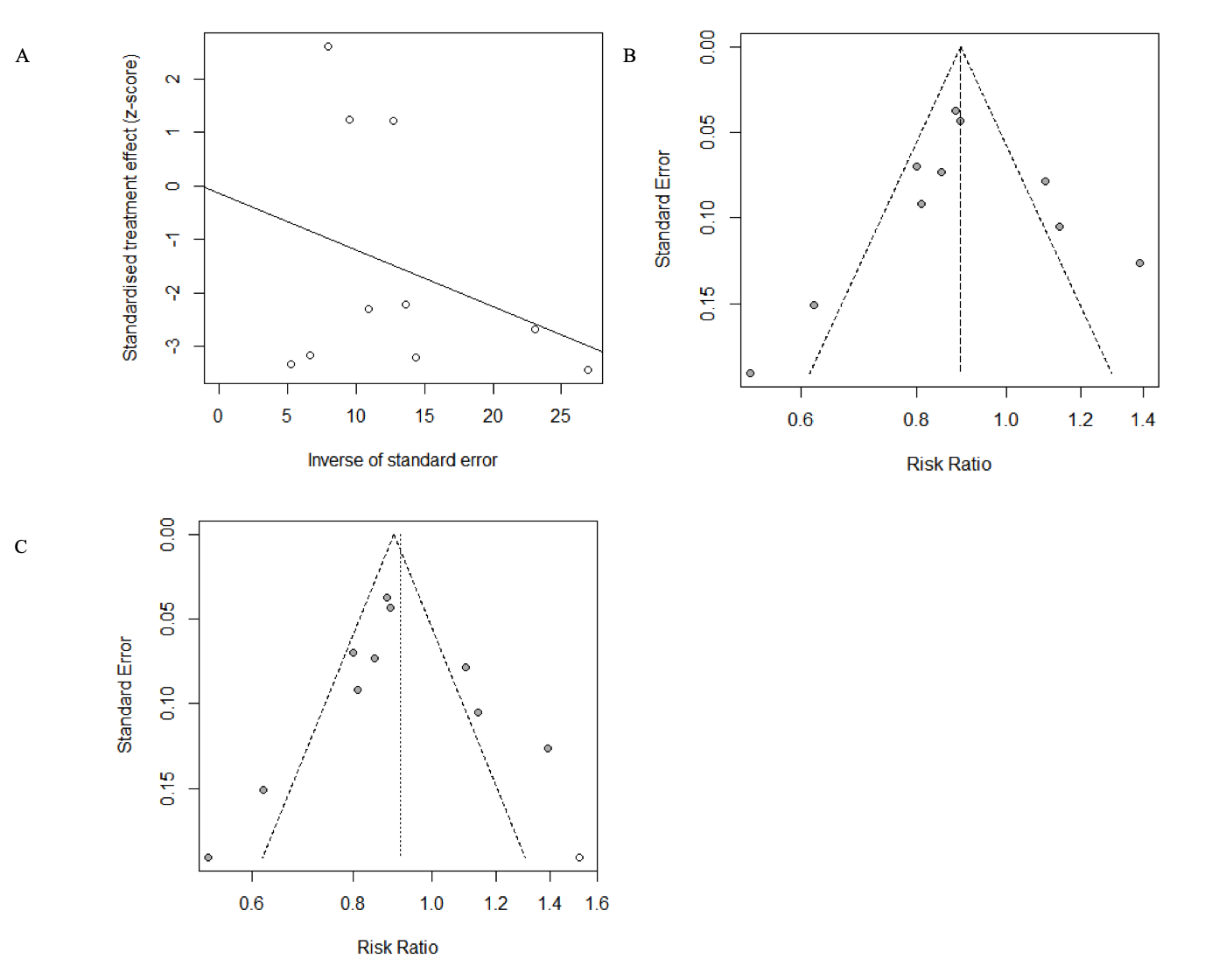


(A) Funnel plot with Egger’s test for the publication bias; (B) Begg’s funnel plot analysis to assess publication bias: Each point represents a separate study included in this meta-analysis; (C) Egger’s funnel plot after “trim-and-fill” procedure to adjust publication bias: Solid circles means study included in this meta-analysis; Hollow circles means one hypothetical unpublished prospective study was added to make the funnel plot more symmetric.

**Supplemental figure S4.** Sensitivity analyses about the influence of single studies on the pooled RR.


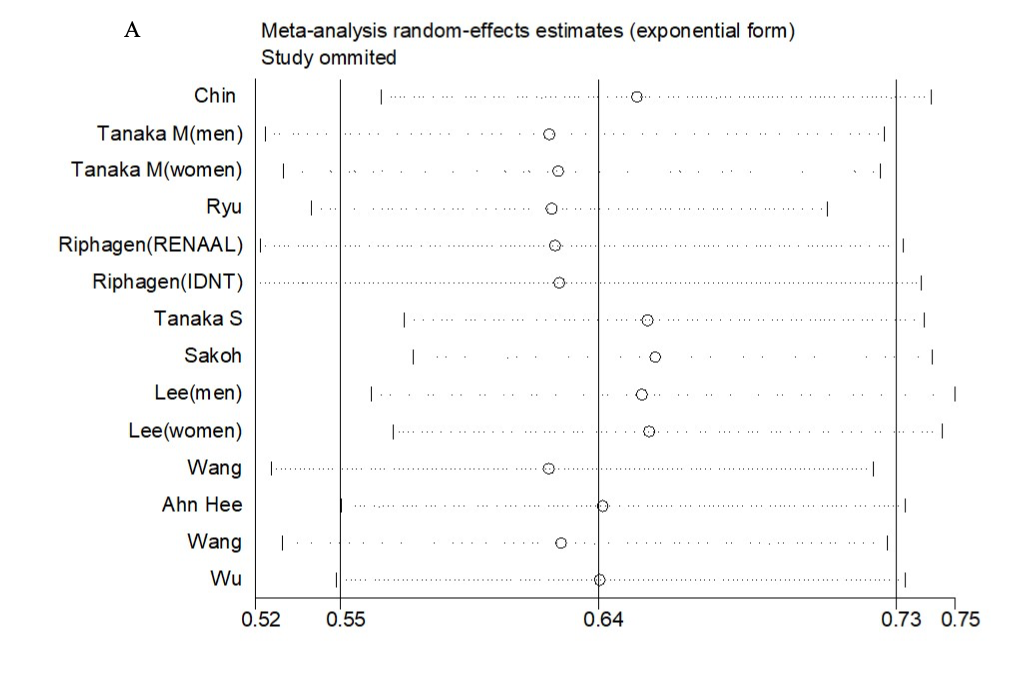


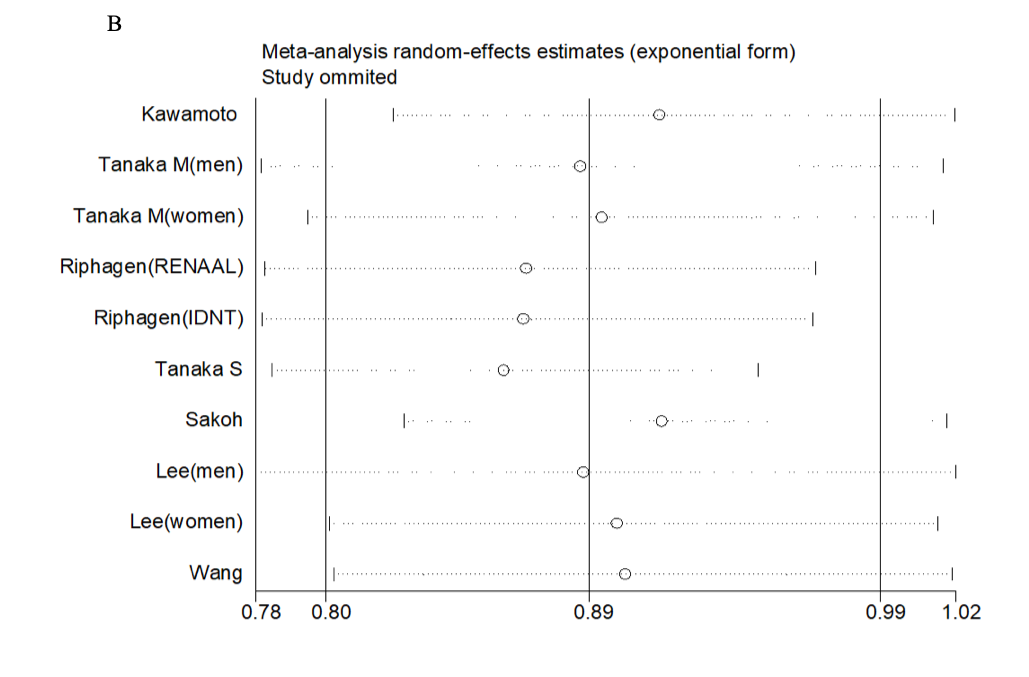


(A) Influence analyses with exclusion of one study at a time on the pooled RR for the association of serum total bilirubin levels (highest to the lowest group) in patients with chronic kidney disease. (B) Influence analyses using per 0.2mg/dL serum total bilirubin increment.

**Supplemental figure S5.** Subgroup and regression analyses of the serum total bilirubin levels and the mortality.


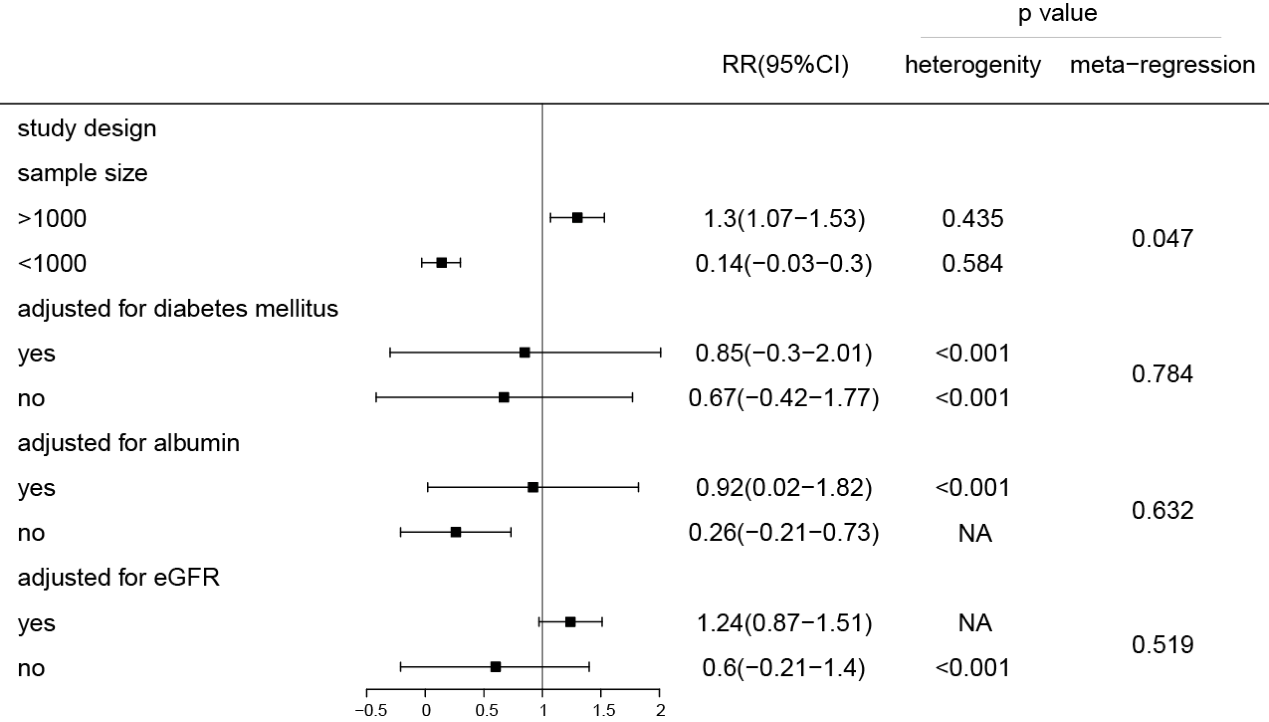


4 studies for the highest compared with the lowest category in serum total bilirubin levels were used within the stratified factors. NA: not applicable; RR: relative risk; CI: confidence interval.
